# Loss of the *Arabidopsis thaliana* P4-ATPases ALA6 and ALA7 impairs pollen fitness and alters the pollen tube plasma membrane

**DOI:** 10.3389/fpls.2015.00197

**Published:** 2015-04-21

**Authors:** Stephen C. McDowell, Rosa L. López-Marqués, Taylor Cohen, Elizabeth Brown, Alexa Rosenberg, Michael G. Palmgren, Jeffrey F. Harper

**Affiliations:** ^1^Department of Biochemistry and Molecular Biology, University of NevadaReno, NV, USA; ^2^Centre for Membrane Pumps in Cells and Disease, Department of Plant and Environmental Sciences, University of Copenhagen, Danish National Research FoundationFrederiksberg, Denmark

**Keywords:** pollen, temperature stress tolerance, lipid flippases, phosphatidic acid, phosphatidylinositol

## Abstract

Members of the P4 subfamily of P-type ATPases are thought to create and maintain lipid asymmetry in biological membranes by flipping specific lipids between membrane leaflets. In Arabidopsis, 7 of the 12 Aminophospholipid ATPase (ALA) family members are expressed in pollen. Here we show that double knockout of ALA6 and ALA7 (*ala6/7*) results in siliques with a ~2-fold reduction in seed set with a high frequency of empty seed positions near the bottom. Seed set was reduced to near zero when plants were grown under a hot/cold temperature stress. Reciprocal crosses indicate that the *ala6/7* reproductive deficiencies are due to a defect related to pollen transmission. *In-vitro* growth assays provide evidence that *ala6/7* pollen tubes are short and slow, with ~2-fold reductions in both maximal growth rate and overall length relative to wild-type. Outcrosses show that when *ala6/7* pollen are in competition with wild-type pollen, they have a near 0% success rate in fertilizing ovules near the bottom of the pistil, consistent with *ala6/7* pollen having short and slow growth defects. The *ala6/7* phenotypes were rescued by the expression of either an ALA6-YFP or GFP-ALA6 fusion protein, which showed localization to both the plasma membrane and highly-mobile endomembrane structures. A mass spectrometry analysis of mature pollen grains revealed significant differences between *ala6/7* and wild-type, both in the relative abundance of lipid classes and in the average number of double bonds present in acyl side chains. A change in the properties of the *ala6/7* plasma membrane was also indicated by a ~10-fold reduction of labeling by lipophilic FM-dyes relative to wild-type. Together, these results indicate that ALA6 and ALA7 provide redundant activities that function to directly or indirectly change the distribution and abundance of lipids in pollen, and support a model in which ALA6 and ALA7 are critical for pollen fitness under normal and temperature-stress conditions.

## Introduction

Biological membranes are highly organized structures and often have a non-random distribution of lipid species between their constituent leaflets (Van Meer, 2011). Members of the P4 subfamily of P-type ATPases (P4-ATPases) have been shown to catalyze the flipping of phospholipids across biological membranes (Coleman et al., 2009; Zhou and Graham, 2009) and are thought to help create and maintain lipid asymmetry between membrane leaflets (Paulusma and Elferink, 2010; Sharom, 2011; Tanaka et al., 2011; Coleman et al., 2013; Hankins et al., 2015; López-Marqués et al., 2015). Lipid asymmetry between membrane leaflets, and its dissipation, have been linked to a wide variety of cellular processes including: cell-to-cell signaling, regulation of membrane permeability, vesicular trafficking, enzyme regulation, and apoptosis (Verhoven et al., 1995; Tannert et al., 2003; Fernandis and Wenk, 2007; Muthusamy et al., 2009; Paulusma et al., 2009; Sebastian et al., 2012; Xu et al., 2013). Studies in yeast and plants have implicated P4-ATPases in vesicular trafficking and tolerance to varied temperature (Ripmaster et al., 1993; Chen et al., 1999; Gomès et al., 2000; Gall et al., 2002; Hua et al., 2002; Pomorski et al., 2003; Poulsen et al., 2008; McDowell et al., 2013). However, the mechanistic relationship between these functions and flippase activity has not been determined.

Evidence indicates that P4-ATPases have different substrate preferences. For example, in yeast, Drs2p transports PS (phosphatidylserine) and PE (phosphatidylethanolamine) (Natarajan et al., 2004; Zhou and Graham, 2009), whereas Dnf1p transports both PC (phosphatidylcholine) and PE (Kato et al., 2002; Pomorski et al., 2003). Although many P4-ATPases require interaction with a CDC50-family protein for ER export and flippase activity, evidence from Arabidopsis shows that subcellular localization and substrate specificity are determined by the P4-ATPase (López-Marqués et al., 2010). Recently, studies of yeast and mammalian P4-ATPases have identified residues that contribute to their substrate specificities (Baldridge and Graham, 2012, 2013; Vestergaard et al., 2014).

The P4-ATPase family in *Arabidopsis thaliana* consists of 12 proteins: ALA 1 to ALA12 (Axelsen and Palmgren, 2001; Baxter et al., 2003; Pedersen et al., 2012). Flippase activity has been reported for ALA2 and ALA3 when co-expressed with a beta-subunit in a yeast mutant deficient for its endogenous plasma membrane (PM) localized P4-ATPases (*dnf1Δdnf2Δ*) (Poulsen et al., 2008; López-Marqués et al., 2010). ALA2 specifically transports PS, whereas ALA3 transports PE, PC, and PS (Poulsen et al., 2008; López-Marqués et al., 2010). Additionally, ALA1 has been shown to localize to the PM (López-Marqués et al., 2012), ALA2 to the PVC (López-Marqués et al., 2010) and ALA3 to the *trans*-Golgi network (Poulsen et al., 2008). Of the 12 ALA isoforms, knockout phenotypes have only been reported for *ala3* mutants (Poulsen et al., 2008; Zhang and Oppenheimer, 2009; McDowell et al., 2013). Loss of ALA3 results in pleiotropic phenotypes affecting root, shoot, and reproductive development. Additionally, *ala3* mutants are highly sensitive to growth conditions such as temperature and soil. A cold-sensitive reduction in plant size has also been observed for plants expressing an RNAi construct against ALA1 (Gomès et al., 2000).

Here, we present evidence that the Arabidopsis P4-ATPases ALA6 and ALA7 are important for rapid, sustained pollen tube growth and are essential for temperature stress tolerance. Genetic evidence indicates that the activities of ALA6 and ALA7 are highly redundant. Pollen defects could be rescued by the expression of fluorescently-tagged ALA6 fusion proteins, which localized simultaneously to both the plasma membrane and highly mobile endomembrane vesicles. Mass spectrometry analysis of mature pollen grains revealed significant differences in lipid composition between *ala6-1/7-2* and wild-type. We also show that the ability of lipophilic FM dyes to stain *ala6-1/7-2* pollen tubes is reduced by ~10-fold relative to wild-type, indicating altered properties associated with the plasma membrane. Together, these results suggest a model in which ALA6 and ALA7 directly or indirectly change the distribution and concentration of lipids in pollen, and that these flippases are critical for pollen fitness under normal and temperature-stress conditions.

## Materials and methods

### T-DNA insertion mutants

**Figure 1:**
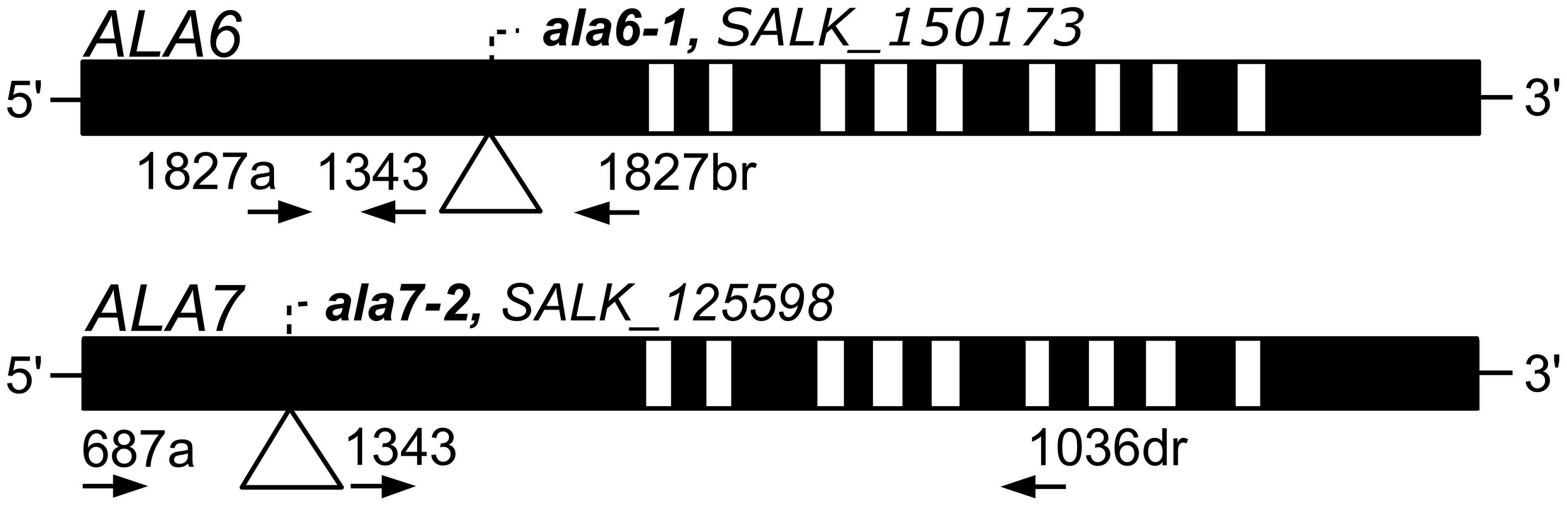
**Diagrams of *ALA6* and *ALA7* showing locations of *T-DNA* disruptions**. Filled boxes represent exons and open boxes represent introns. *T-DNA* insertions are represented with triangles and identified by *ala* allele numbers and *T-DNA* allele accessions. Primers used for PCR genotyping are represented by arrows and point in the 5′ to 3′ direction. Primer 1343 corresponds to the *T-DNA* left border. The left-border junctions are as follows: *ala6-1*, TGGGACTCCGGCTCAAGCACGCACCgatcgccttaatcgccttaatccgt; and *ala7-2* LB: atttgtttacaccacaatatatcctGAACTATCAAATGTGAAGATCCCAA. Capital letters represent *ALA* genomic DNA and lowercase letters represent *T-DNA*.

Two T-DNA insertional mutants were used in this study: *ala6-1* (SALK_150173, ss757) and *ala7-2* (SALK_125598, ss733) (Alonso et al., 2003). Mutants were obtained from the Arabidopsis Biological Resource Facility at Ohio State University (http://abrc.osu.edu/) and were identified using PCR-based screening techniques. Both mutants are in the Col-0 background. The locations of the T-DNA insertions and PCR primers are indicated in Figure 1. Sequences for the PCR primers can be found in File S2. The individual mutant lines were crossed to create the double mutant *ala6-1/7-2* (ss1351).

### Plant growth conditions

Seeds were sown on 0.5× Murashige and Skoog medium (pH 5.7) containing 1% agar and 0.05% MES. Following 48 h of stratification (4°C, dark), seedlings were grown at room temperature (~23°C) under 24 h light for 7–10 d before being transplanted to soil. The soil used was Sunshine SMB-238 (SunGro Horticulture, Agawam, MA) supplemented with 10-10-10 fertilizer and Marathon pesticide following the manufacturer's instructions. Plants were grown until maturity in a growth chamber (Percival Scientific, Perry, IA) under a long-day photoperiod (16 h light at 20°C/8 h dark at 18°C, 70% humidity, and ~125 μmol m^−2^ s^−1^ light intensity).

### Plasmid construction

For expression in plants, two ALA6 genomic DNA fragments were PCR amplified from BAC clone F20D21 using the primer pairs 1035a+br (C-terminal tag) and 1035a+brs (N-terminal tag) and Phusion High-Fidelity DNA Polymerase (New England Biolabs, Ipswich, MA). An ALA1 genomic DNA fragment was amplified from Arabidopsis genomic DNA (Col-0 ecotype) using the primer pair 1030a+br (C-terminal tag) and Pfu-Turbo DNA Polymerase (Agilent, Santa Clara, CA). PCR primer sequences can be found in File S2. Poly-A tails were added to the PCR products using ExTaq (Takara, Mountain View, CA) and the resultant DNA fragments were T/A cloned into the pGEM-T Easy vector (Amp^r^ in bacteria) using the pGEM-T Easy Vector System kit (Amp^r^ in bacteria) (Promega, Madison, WI). All PCR-derived fragments were sequence verified to be error free in the pGEM-T Easy vector. The ALA6 and ALA1 fragments were subcloned from the pGEM vector into a derivative of the pGreenII plant vector (kan^r^ in bacteria, hyg^r^ in plants) (Hellens et al., 2000). Junction sites were sequence verified to be error free. All fusion proteins expressed in this study were under the control of the *Arabidopsis thaliana ACA9* promoter, which drives a moderate expression preferentially in pollen (Schiøtt et al., 2004). Internal stock numbers for each plasmid are: ps1730, ACA9p-NTAP2(G)-ALA6; ps1728, ACA9p-i-ALA6-TAP2(Y); ps1729, ACA9p-i-ALA1-TAP2(Y); and ps779, ACA9p-i-TAP2(Y). Sequence information for each plasmid can be found in File S3.

For expression in yeast, two ALA6 cDNA fragments were amplified using the primer pairs oli4300+oli4301 (untagged ALA6) and oli4299+oli4301 (RGSH10-ALA6). PCR primer sequences can be found in File S2. The ALA6 cDNA template was created from total RNA extracted from Col-0 pollen. The PCR fragments were cloned into the yeast plasmids pRS423-GAL (untagged) (Burgers, 1999) and pMP4062 (His tag, RGSH10) (López-Marqués et al., 2012) using homologous recombination. PCR fragments and corresponding plasmids were transformed into the *S. cerevisiae* strain ZHY709 (*MATα his3 leu2 ura3 met15 dnf1Δ dnf2Δ drs2::LEU2*) (Hua et al., 2002) using the lithium acetate method (Gietz and Woods, 2002). Positive transformants were identified after 4 d of growth at 28°C using synthetic complete media (SCD) plates without histidine (0.7% Yeast Nitrogen base, 2% glucose, 1× drop out media supplement) (Rose and Broach, 1990). All PCR-derived fragments were sequence verified to be error free.

### Plant transformation

Plants were transformed with *Agrobacterium tumefaciens* strain GV3101 carrying the pSOUP helper plasmid using the floral dip method (Clough and Bent, 1998; Hellens et al., 2000). T1 seedlings were grown on 0.5× Murashige and Skoog (MS) medium (pH 5.7) containing 1% agar, 0.05% MES, and 25 μg/ml hygromycin to identify successful transformants.

### *In-vitro* pollen tube growth

The pollen tube growth medium was based on the method described by Boavida and McCormick, and contained: 5 mM CaCl_2_, 0.01% H_3_BO_3_, 5 mM KCl, 10% sucrose, 1 mM MgSO_4_, pH 7.5–7.8, and 1.5% low melting agarose (Boavida and McCormick, 2007). Pollen from stage 13 to 14 flowers was placed on pistils, either from the corresponding genotype or from surrogate *ms-1* plants, and the pistils were then placed on ~400 μL of pollen tube growth medium layered over a microscope slide. The slides were incubated at room temperature (~23°C) in a square petri dish containing water-soaked paper towels to maintain high humidity. Pollen tubes were grown for 2–6 h prior to analysis, unless being used for a time course. For the time course analysis of pollen tube length, pollen tubes were photographed with a Hamamatsu Orca ER camera attached to a Leica DM-IRE2 microscope (JH Technologies, Fremont, CA). Length measurements were done using the Fiji software package (Schindelin et al., 2012).

### Confocal microscopy

Images were collected using an Olympus IX81 FV1000 confocal microscope run by the Olympus FluoView 1.07.03.00 software package (Olympus, Center Valley, PA). A 60× objective (numerical aperture 1.42) was used throughout. Excitation at wavelengths of 488 nm (GFP, FM4-64, and FM1-43) and 515 nm (YFP) was provided with an Argon-Ion laser. A spectral emission range of 500–600 nm was used for GFP, 545–595 nm for YFP, 670–726 nm for FM-4-64, and 575–605 nm for FM1-43.

### FM dye staining

Pollen tubes were stained with the lipophilic dyes FM4-64 and FM1-43 (Invitrogen - Molecular Probes, Eugene, OR). For both dyes, 10 μM staining solutions were prepared by dissolving the individual dye in liquid pollen tube growth medium. When needed, sodium azide (NaAz) was added to the staining solution to a final concentration of 0.05%. Staining solution was directly applied to pollen tubes growing on solid medium layered over a microscope slide (see above section: *In-vitro* Pollen Tube Growth). Images were captured 1–45 m after the addition of the staining solution, as described in the Confocal Microscopy Section. Fluorescence was quantified in terms of average pixel intensity using the Fiji software package (Schindelin et al., 2012).

### Lipid profiling

Pollen for lipid analysis was collected from independent, parallel-grown groups of ~75 plants. Total lipid extracts were obtained from pollen using chloroform/methanol extraction, described below. To deactivate phospholipases prior to lipid extraction, pollen samples were immersed for 15 m in 3 mL of 75°C isopropanol + 0.01% butylated hydroxytoluene (BHT). The first extraction step was done by adding 1.5 mL chloroform and 0.6 mL of water to the pollen/isopropanol mixture. Four subsequent extraction steps were done using chloroform/methanol (2:1) + 0.01% BHT. Each extraction was done for 1 h; except for the last extraction, which was done overnight (~12 h). The five extracts for each sample were combined and washed twice: first with 1 mL of 1 M KCl and second with 2 mL of water.

Lipid samples were then evaporated to 1 mL and sent to the Kansas Lipidomics Research Center (http://www.k-state.edu/lipid/lipidomics) for routine plant polar lipid analysis by tandem mass spectrometry. The two mass spectrometers used were an Applied Biosystems API 4000 and an Applied Biosystems Q-TRAP, separated by a collision cell. Samples were introduced by electrospray ionization, with no pre-analysis separation. Analysis was done using both precursor and neutral loss scans.

### Lipid translocation assays

Lipid translocation was assayed as previously described (López-Marqués et al., 2010). ALA6-containing yeast (see Plasmid Construction) was transformed with the yeast plasmid pRS426-GAL (Burgers, 1999); either empty, or containing the putative beta-subunits ALA interacting subunit 1 (ALIS1), ALIS3, or ALIS5 (Poulsen et al., 2008). As controls, the wild type BY4741 (*MATα his3 leu2 ura3 met15*; EUROSCARF) (positive control) and the mutant ZHY709 (negative control) were transformed with empty plasmids pRS423-GAL and pRS426-GAL. Flow cytometry was performed on a FACSCalibur cell analyzer (BD Biosciences, San Jose, CA) equipped with an argon laser using Cell Quest software. Thirty thousand cells were analyzed without gating during the acquisition, and live cells were selected based on forward/side-scatter gating and propidium iodide exclusion. A histogram of the green (NBD) fluorescence of living cells was used to calculate the mean fluorescence intensity of total cells.

### Yeast membrane fractionation and immunolabeling

Fractionation of yeast membranes in sucrose gradients, quantification of protein contents, and Western blot analysis were carried out as described in López-Marqués et al. (2012). Fractions enriched in ER (30% sucrose) and PM (48% sucrose) were collected by sucrose density gradient fractionation and used for Western blot analysis. Immunodetection of RGSH6- and RGSH10-tagged proteins was performed using a commercial BSA-free RGS-His™ antibody produced in mouse (Qiagen, Valencia, CA). We used a monoclonal anti-dolichol phosphate mannose synthase (Dpm1p) antibody (Molecular Probes, Eugene, OR) as a marker for the endoplasmic reticulum and a polyclonal antibody against the C-terminal end of the yeast proton ATPase (Pma1p) (Monk et al., 1991) as a marker for the plasma membrane. Golgi fractions were detected with an affinity purified anti-Sed5p polyclonal antibody (Sapperstein et al., 1996).

## Results

Among the seven ALAs that are most highly expressed in *A. thaliana* pollen, *ALA6* (At1g54280) and *ALA7* (AT3g13900) are two of the most closely related (89% amino acid identity) and account for approximately 56% of the *ALA* subfamily mRNA in pollen grains or growing tubes (Loraine et al., 2013) (Figure S1a). To determine if these genes have redundant functions in pollen development, we isolated the *ala6-1* and *ala7-2 T-DNA* gene disruption lines from the SALK collection (Alonso et al., 2003) and crossed them to make the double knockout *ala6-1/7-2* (Figure 1). The T-DNA insertions for both *ala6-1* and *ala7-2* are in the first exon and are predicted to disrupt the production of functional proteins.

### ALA6 and ALA7 are important for pollen fitness

A pollen transmission defect was observed for double knockout combinations of *ala6-1* and *ala7-2* in which only one allele was segregating (e.g., *ala6-1(–/–)/ala7-2(+/–)* or *ala6-1(+/–)/ala7-2(–/–)*) (Table 1). In plants allowed to self-fertilize, double-homozygous progeny were observed at frequencies of 5.3–8.3%, representing a ~4-fold decrease from the expected 25%. In pollen outcrosses, transmission of the *ala6-1/7-2* allele was reduced to 2.9–3.7%, representing a ~15-fold decrease from the expected 50%. Transmission of *ala6-1/7-2* through the female gametophyte appeared normal, indicating that the observed segregation distortion was the result of a pollen-autonomous defect.

**Table 1:** **Segregation analysis shows a defect in transmission through male gametophytes carrying *ala6-1* and *ala6-1/7-2* mutations**.

**♂ × ♀**	**Cross Description**	**Assay**	**Expected (%)**	**Observed (%)**	***n***	***p*-value**
*ala6-1(+/–)/ala7-2* × same	Selfed	*ala6(–/–)*	25	5.3	94	<0.0001
*ala6-1/ala7-2(+/–)* × same	Selfed	*ala7(–/–)*	25	8.3	144	<0.0001
*ala6-1/ala7-2(+/–)* × WT	Male Outcross	*ala7(–)*	50	3.7	301	<0.0001
WT × *ala6-1/ala7-2(+/–)*	Female Outcross	*ala7(–)*	50	44.7	152	0.1944
*ala6-1(+/–)* × same	Selfed	*ala6(–/–)*	25	20	90	0.4803
*ala7-2(+/–)* × same	Selfed	*ala7(–/–)*	25	27.3	132	0.5465
*ala6-1(+/–)* × WT	Male Outcross	*ala6(–)*	50	25.6	238	<0.0001
*ala7-2(+/–)* × WT	Male Outcross	*ala7(–)*	50	50.2	221	0.9464

*WT is an abbreviation for wild-type. The observed results were compared to an expected Mendelian segregation. Statistical significance was determined by the Pearson's Chi-Squared test*.

Plants heterozygous for *ala6-1(+/–)* or *ala7-2(+/–)* individually showed no evidence of a segregation distortion when allowed to self-fertilize (Table 1). However, manual pollen outcrosses showed a ~2-fold decrease in *ala6-1* transmission, indicating the loss of ALA6 alone can result in a detectable phenotype. A similar deficiency was not seen for *ala7-2*.

Evidence that the pollen transmission defects were caused by loss of ALA6 and ALA6/7 was corroborated by rescuing mutant pollen with transgenes encoding either ALA6-YFP, or GFP-ALA6 (Table 2). Both transgenes were expressed under the control of the *ACA9* promoter, which drives a moderate level of expression preferentially in pollen (Schiøtt et al., 2004). The transgenes were stably expressed in either an *ala6-1* or an *ala6-1/7-2* mutant background, and reciprocal crosses were done to test for an increase in the transmission efficiency of gametes harboring a transgene. Both transgenes showed an expected 50% transmission through the female, confirming that only one copy of the transgene was present in the plants used in the reciprocal crosses. In contrast, pollen transmission of the transgenes was increased to 78–83% in the *ala6-1* background and 98–99% in the *ala6-1/7-2* background. Neither transgene showed an increased pollen transmission in wild-type control plants, indicating that the improved transmission in mutants was the result of rescuing the pollen defects associated with *ala6-1* and *ala6-1/7-2* knockouts. Additional controls showed that *ala6-1/7-2* pollen was not rescued by an empty vector or a more distantly related ALA isoform, ALA1. Since the transmission defect associated with the double mutant was more pronounced, subsequent studies were done using *ala6-1/7-2*.

**Table 2:** **Segregation analysis shows that the pollen transmission defects of *ala6-1* and *ala6-1/7-2* can be rescued by ALA6-YFP or GFP-ALA6 fusion proteins**.

**♂ × ♀**	**Cross Description**	**Transgene (TG)**	**Expected TG (%)**	**Observed TG (%)**	***n***	***p*-Value**
*ala6-1/7-2* + TG(+/*–*) × same	Selfed	ALA6-YFP, GFP-ALA6	75	93.2, 91.5	869, 1509	Both <0.0001
*ala6-1/7-2* + TG(+/*–*) × WT	Male Outcross	ALA6-YFP, GFP-ALA6	50	97.5, 99.1	475, 875	Both <0.0001
*ala6-1* + TG(+/*–*) × WT	Male Outcross	ALA6-YFP, GFP-ALA6	50	77.7, 82.8	282, 331	Both <0.0001
WT × *ala6-1/7-2* + TG(+/*–*)	Female Outcross	ALA6-YFP, GFP-ALA6	50	51.7, 51.3	487, 567	0.4411, 0.5287
WT × *ala6-1* + TG(+/*–*)	Female Outcross	ALA6-YFP, GFP-ALA6	50	51.4, 51.6	140, 221	0.7353, 0.6377
*ala6-1/7-2* + TG(+/*–*) × same	Selfed	YFP	75	72.5	1601	0.0218
*ala6-1/7-2* + TG(+/*–*) × same	Selfed	ALA1-YFP	75	75.6	324	0.7975
WT + TG(+/*–*) × same	Selfed	ALA6-YFP, GFP-ALA6	75	68.3, 71.4	1854, 2735	Both <0.0001

*Lines containing a single copy of the transgene were maintained through outcrosses. WT is an abbreviation for wild-type and TG is an abbreviation for transgene. The observed results were compared to an expected Mendelian segregation. Statistical significance was determined by the Pearson's Chi-Squared test. For the GFP-ALA6 rescue construct: 6/6 lines showed equivalent rescue of ala6-1/7-2 (ss1878–1881, ss1883–1884); 3/3 lines showed equivalent rescue of ala6-1 (ss1889–1891); and 9/9 lines did not improve reproductive fitness in WT (ss1895–1903). For the ALA6-YFP rescue construct, 3/3 lines showed equivalent rescue of ala6-1/7-2 (ss1885, ss1886, ss1888); 3/3 lines showed equivalent rescue of ala6-1 (ss1892–1894); and 6/6 failed to show any changes to reproductive fitness in a WT background (ss1904–1909). For the YFP-only control construct, 7/7 lines failed to rescue ala6-1/7-2 (ss1910–1916). For the ALA1-YFP control construct, 2/2 lines failed to rescue ala6-1/7-2 (ss1917–1918)*.

### Seed set in *ala6-1/7-2* is reduced and hypersensitive to temperature stress

**Figure 2:**
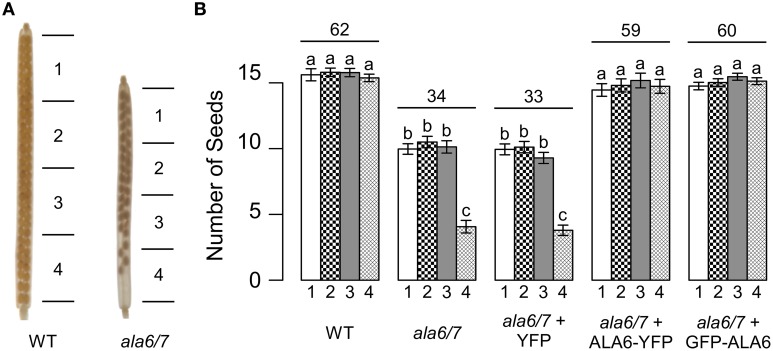
**Loss of *ALA6* and *ALA7* results in reduced seed set with an uneven distribution of seed**. **(A)** Representative examples of wild-type and *ala6-1/7-2* siliques cleared with 70% EtOH to show seed positions. **(B)** Graph of seed set by sector. Siliques were divided into four sectors of equal length with sector 1 at the top (stigma end) of the silique and sector 4 at the base of the silique. Average results (±SE) are reported for two independent experiments, *n* = 35–93 siliques. Siliques were collected from a total of 5–13 plants for each genotype. Sector numbers appear below each column and the average total seed set for each genotype is given above the corresponding sector data. For the GFP-ALA6 rescue construct, 7/9 lines showed equivalent rescue of *ala6-1/7-2*: ss1878–1884. For the ALA6-YFP rescue construct, 4/6 lines showed equivalent rescue of *ala6-1/7-2:* ss1885–1888. *^a,b,c^*Columns sharing common labels (letters) are not significantly different from each other (*p* > 0.05).

In homozygous *ala6-1/7-2* mutants, seed set within each silique was decreased to ~55% that of wild-type (Figure 2). Furthermore, seed was unevenly distributed within *ala6-1/7-2* siliques, with a high frequency of empty seed positions at the bottom of the silique. Seed set was restored to wild-type levels in *ala6-1/7-2* plants expressing a GFP-ALA6 or an ALA6-YFP construct, but not an empty vector control, indicating that the phenotype was caused by loss of ALA6 and ALA7. A seed set phenotype was not observed when *ala6-1/7-2* pistils were manually fertilized with wild-type pollen (Figure S2), indicating that pollen defects alone account for the mutant phenotype.

**Figure 3:**
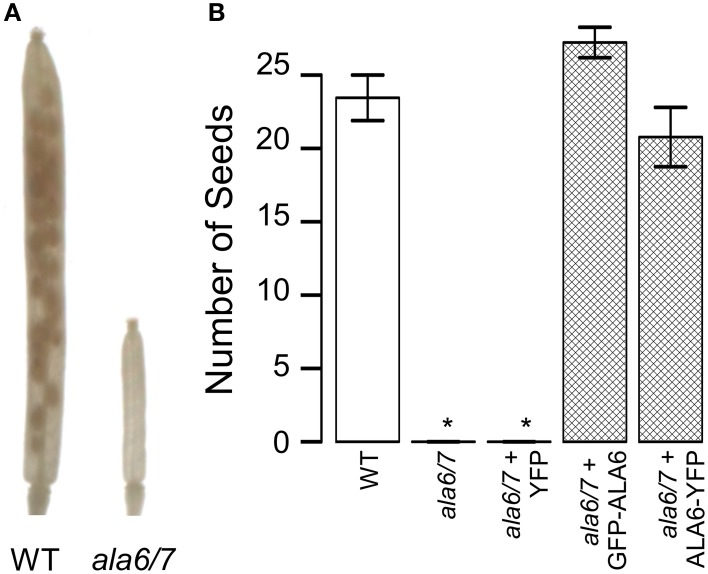
**Loss of ALA6 and ALA7 results in sterility under hot-day/cold-night temperature stress**. Plants were allowed to self-fertilize under a temperature stress that cycled between hot-days (40°C peak) and cold-nights (−1°C low) with periods of intermediate temperature between the extremes for acclimation. **(A)** Representative examples of wild-type and *ala6-1/7-2* siliques cleared with 70% EtOH to show seed positions. **(B)** Graph of overall seed set. Average results (±SE) are reported for two independent experiments, *n* = 15–85 siliques. Siliques were collected from at least four plants for each genotype. For the GFP-ALA6 rescue construct, 6/6 lines showed equivalent rescue of *ala6-1/7-2*: ss1878, ss1879, and ss1881-1884. For the ALA6-YFP rescue construct, 3/3 lines showed equivalent rescue of *ala6-1/7-2:* ss1885, ss1887, and ss1888. ^*^Statistically significant difference between wild-type and *ala6-1/7-2* (Welch's *t*-test, *p* < 0.05).

To determine if the reduction in *ala6-1/7-2* seed set is dependent upon growth conditions, plants were allowed to self-fertilize under a temperature stress that cycled between hot days (40°C peak) and cold-nights (−1°C low) (Figure S3). The temperature stress caused seed set in wild-type siliques to be reduced to ~38% that of unstressed, whereas *ala6-1/7-2* mutants were sterile (Figure 3). Seed set was restored to wild-type levels in *ala6-1/7-2* plants expressing a GFP-ALA6 or an ALA6-YFP construct, but not an empty vector control, confirming that the sensitivity to temperature stress was caused by loss of ALA6 and ALA7.

### *Ala6-1/7-2* pollen tubes are short and slow

**Figure 4:**
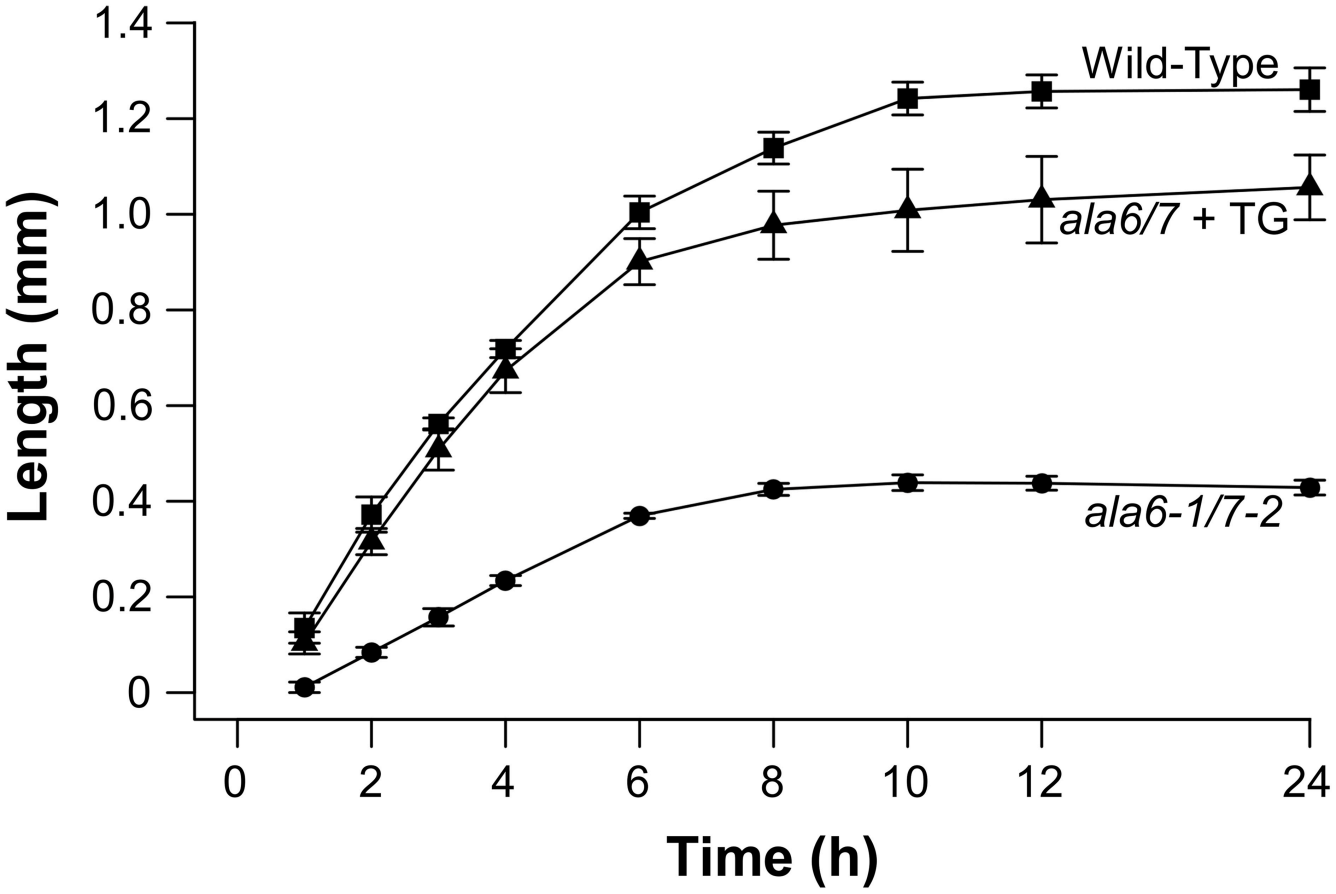
***In-vitro* assays show *ala6-1/7-2* pollen tubes are short with reduced rates of growth**. Pollen was placed on pistils, either from the corresponding genotype or from surrogate *ms-1* plants, and the pistils were placed on agar-solidified growth media. Pollen tubes growing out of the pistils were measured over a 24 h time course. Lengths were reported for each time point as the average length of the 10 longest pollen tubes. Values and error bars represent mean ± SE for three independent experiments for all genotypes. An independent transgenic line was used for each *ala6-1/7-2* + TG experiment (ss1883, ss1886, and ss1888) with each line showing similar results.

To quantify growth defects associated with *ala6-1/7-2* pollen, *in-vitro* growth assays were done over a 24 h time course (Figure 4). After the 24 h growth period, the overall length of *ala6-1/7-2* pollen tubes was ~35% that of wild-type. The maximal growth rate of *ala6-1/7-2* pollen tubes was also reduced to ~45% that of wild-type. Expression of two different *ALA6* transgenes (*ALA6-YFP* and *GFP-ALA6*) restored the growth rate and overall length of *ala6-1/7-2* pollen tubes to nearly that of wild-type.

To evaluate the *in-vivo* relevance of *ala6-1/7-2* pollen tube growth defects, pollen from *ala6-1(–/–)/7-2(+/–)* plants was used to fertilize wild-type pistils, and the resulting mature siliques were divided into three sectors of equal length (top, middle, and bottom). Without growth defects, the *ala6-1/7-2* allele would be expected to transmit to all three sectors equally, with 33% of the total transmission in each sector. However, 72.7% of the *ala6-1/7-2* pollen transmission was observed in the top sector, whereas no transmission of *ala6-1/7-2* was observed in the bottom sector (Table 3). These results indicate that the competitive fitness of *ala-16/7-2* pollen relative to wild-type decreases in the distal region of the pistil, consistent with *in-vitro* growth assays showing *ala6-1/7-2* pollen tubes to be slow and short (Figure 4).

**Table 3:** **The transmission of *ala6-1/7-2* through pollen is restricted to the top 2/3 of the silique**.

**♂ × ♀**	**Assay**	**% Total *ala6-1/7-2* Transmission**			***n***	***p*-Value**
		**Top**	**Middle**	**Bottom**		
Expected	n/a	33	33	33	n/a	n/a
*ala6-1/7-2(+/–)* × WT	*ala7(–)*	72.7	27.3	0	11	0.01

*Wild-type (WT) and ms-1 pistils were fertilized with ala6-1(–/–)/7-2(+/–) pollen and the resulting siliques were divided into three sectors of equal length Top (stigma end), Middle, and Bottom (base of the silique). The observed results are compared to an expected equal distribution of mutant alleles across all three sectors. Statistical significance was determined by the Pearson's Chi-Squared test*.

### The subcellular localization of ALA6 includes the plasma membrane and endomembrane structures

**Figure 5:**
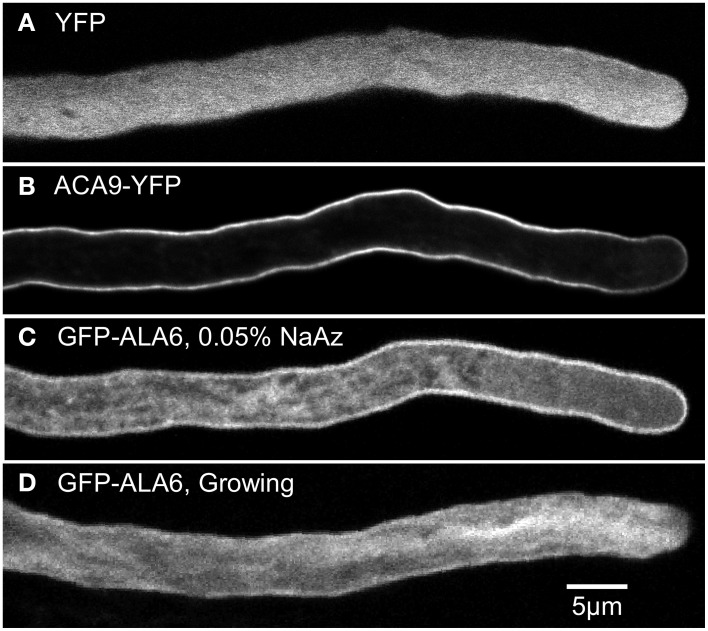
**Confocal fluorescence micrographs showing GFP-ALA6 localizes to the pollen tube perimeter and endomembrane structures**. **(A,B)** Growing pollen tubes expressing **(A)** YFP (ss1919) as a marker for the cytosol and **(B)** ACA9-YFP (ss471-472) as a marker for the plasma membrane (Myers et al., 2009). **(C,D)** Pollen tubes expressing GFP-ALA6 (ss1880) either **(C)** treated with 0.05% NaAz, or **(D)** growing. Constructs were expressed under the control of the ACA9 promoter in stable transgenic Arabidopsis plants. Images of GFP-ALA6 are representative of seven GFP-ALA6 (ss1878–1884) and four ALA6-YFP (ss1885–1888) transgenic lines in which the transgenes were shown to rescue the *ala6-1/7-2* phenotype. The pattern of localization was equivalent for expression levels that ranged from high to the lower limits of detection. The images shown for GFP-ALA6 represent GFP signals that were significantly above background autofluorescence, as determined by comparison with WT pollen imaged using the same exposure settings (black images not shown).

The potential subcellular localization of ALA6 was investigated using confocal microscopy to image GFP-ALA6 and YFP-ALA6 fusion proteins in the pollen tubes of stable *ala6-1/7-2* transgenic plants (Figure 5). The transgenes encoding both fusion proteins were shown to rescue *ala6-1/7-2* pollen defects (Figures 2, 3; Table 2). In pollen tubes treated with 0.05% NaAz, both ALA6 fusion proteins showed a localization pattern consistent with a plasma membrane association, although some association with endomembrane structures was also observed (Figure 5C, Movie S1). In growing pollen tubes not treated with NaAz, the relative amount of endomembrane-associated ALA6 fusion protein was higher, with the ALA6-labeled structures engaged in cytoplasmic streaming (Figure 5D, Movie S2). These localization patterns were observed for 11 of 11 independent transgenic lines (7 GFP-ALA6, 4 ALA6-YFP), regardless of whether expression levels were high or at the lower limits of detection, providing evidence that the patterns were not associated with an over-expression artifact.

### Lipid composition is altered in *ala6-1/7-2* pollen grains

**Figure 6:**
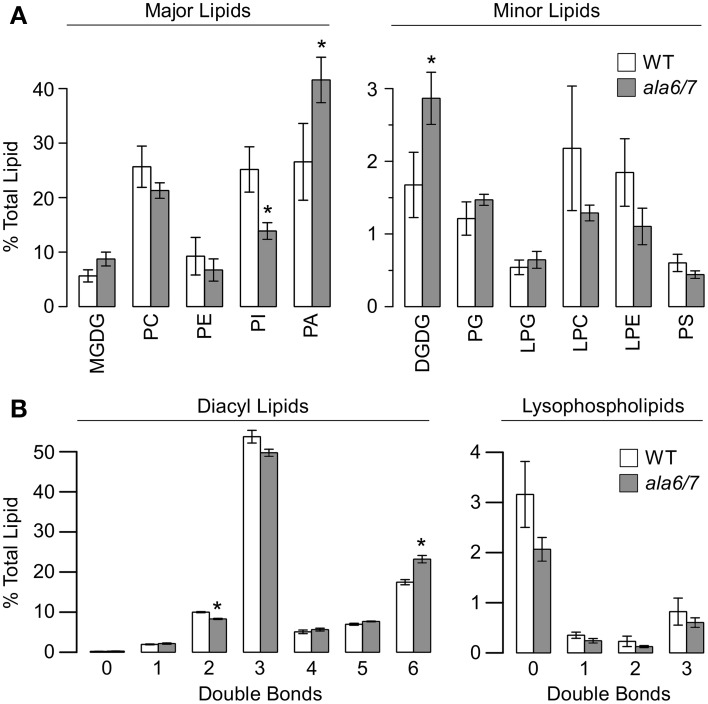
**The quantification of lipid head-groups and unsaturation reveals significant differences between wild-type and *ala6-1/7-2* pollen**. Lipid concentrations were measured using tandem mass spectrometry (MS/MS) that detected 11 different head-groups and quantified the acyl carbons and double bonds within the corresponding acyl side chain(s). Concentrations are expressed as a percentage of the total lipid detected for a specific sample and are represented as mean ± SE, *n* = 4 for both WT and *ala6-1/7-2*. Pollen was collected from independent, parallel-grown, groups of ~75 plants. **(A)** Concentrations of lipid head-groups. Higher- concentration head-groups appear on the left and lower-concentration head-groups appear on the right. **(B)** Unsaturation in acyl side chain(s). Unsaturation in diacyl lipids (2 acyl chains) appears on the left and unsaturation in lysophospholipids (1 acyl chain) appears on the right. Abbreviations: MGDG, monogalactosyldiacylglycerol; PC, phosphatidylcholine; PE, phosphatidylethanolamine; PI, phosphatidylinositol; PA, phosphatidic acid; DGDG, digalactosyldiacylglycerol; PG, phosphatidylglycerol; LPG, lysophosphatidylglycerol; LPC, lysophosphatidylcholine; LPE, lysophosphatidylethanolamine; PS, phosphatidylserine. ^*^Statistically significant difference between wild-type and *ala6-1/7-2* (Welch's *t*-test, *p*<0.05).

To determine if loss of ALA6/7 is correlated with a change in lipid composition, tandem mass spectrometry (MS/MS) was done on lipids extracted from *ala6-1/7-2* and wild-type pollen grains (Figure 6, File S1). The MS/MS analysis detected polar lipids from 11 head-groups (MGDG, monogalactosyldiacylglycerol; PC, phosphatidylcholine; PE, phosphatidylethanolamine; PI, phosphatidylinositol; PA, phosphatidic acid; DGDG, digalactosyldiacylglycerol; PG, phosphatidylglycerol; LPG, lysophosphatidylglycerol; LPC, lysophosphatidylcholine; LPE, lysophosphatidylethanolamine; and PS, phosphatidylserine) and quantified the double bonds within the corresponding acyl side chain(s). In total, the abundances of 144 distinct lipids were measured. We chose to examine pollen grains instead of growing tubes because pollen grains could be more easily harvested in sufficient quantities, with the majority of cells in the same developmental and physiological state. Expression profiling data suggests that both ALA6 and ALA7 are expressed at similar levels in mature pollen grains and growing pollen tubes (Figure S1b). Significant differences between *ala6-1/7-2* and wild-type were observed, both in the concentrations of lipid head-groups (Figure 6A) and the average number of double bonds (i.e., unsaturation) within acyl side chains (Figure 6B). For example, we observed a ~2-fold decrease in PI concentration and a ~2-fold increase in PA concentration in *ala6-1/7-2* pollen relative to wild-type.

### FM-dye staining is reduced in the *ala6-1/7-2* pollen tube plasma membrane

**Figure 7:**
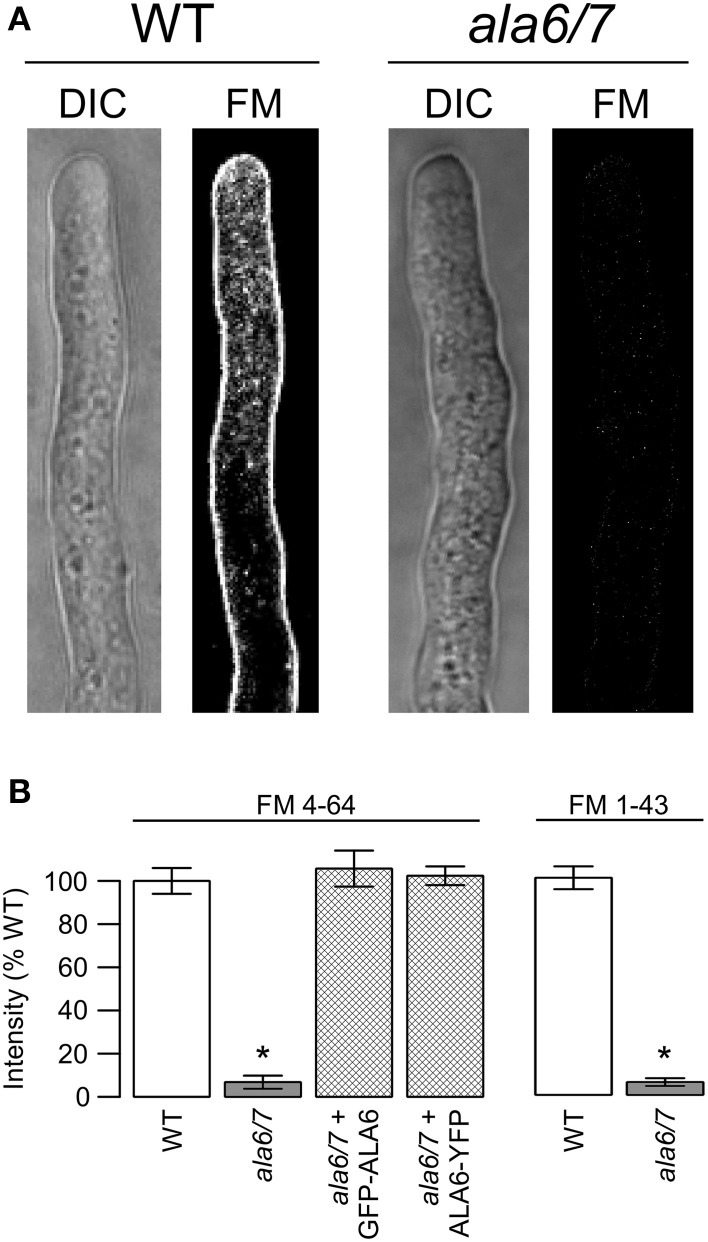
**Loss of ALA6 and ALA7 reduces the ability of lipophilic FM dyes to stain living pollen tubes**. **(A)** Confocal micrographs showing DIC and FM dye emission for wild-type and *ala6-1/7*-2 pollen tubes stained with FM4-64. **(B)** Quantitative analysis of FM dye emission from pollen tubes stained with FM4-64 and FM1-43. Average results (±SE) are reported for three independent experiments, *n* = 21–36 pollen tubes. Pollen tubes were collected from a total of 5–11 different plants for each genotype and dye. For the *GFP-ALA6* rescue construct, 4/4 lines showed equivalent rescue of *ala6-1/7-2*: ss1878, ss1880, ss1881, ss1883. For the *ALA6-YFP* rescue construct, 4/4 lines showed equivalent rescue of *ala6-1/7-2:* ss1885–1888. ^*^Statistically significant difference between wild-type and *ala6-1/7-2* (Welch's *t*-test, *p* < 0.05).

In an attempt to measure rates of endocytosis, wild-type and *ala6-1/7-2* pollen tubes were stained with the lipophilic dyes FM4-64 and FM1-43 (Figure 7). For wild-type tubes only, both FM dyes showed strong PM staining after less than 2 m, and increasing internal staining over a 45 m time period. In contrast, *ala6-1/7-2* pollen tubes failed to show any significant PM or internal staining, even after 45 m. However, both wild-type and *ala6-1/7-2* pollen tubes showed similar staining when exposed to the sterol-dye Filipin (Boutté et al., 2011) (Figure S4a), indicating that the change in the *ala6-1/7-2* plasma membrane that limits FM-dye staining does not affect all dyes. Additionally, the FM dyes were able to stain membranes throughout *ala6-1/7-2* pollen tubes that had been killed by prolonged NaAz exposure (Figure S4.3b), indicating that loss of ALA6 and ALA7 does not affect all membranes equally.

## Discussion

### ALA6 and ALA7 are critical for rapid, sustained pollen tube growth

In this study, we present genetic evidence that the simultaneous loss of ALA6 and ALA7 result in a male-autonomous reproductive defect (Tables 1, 2). *In-vitro* growth assays indicate that, relative to wild-type, *ala6-1/7-2* pollen tubes have more than 2-fold reductions in both maximal growth rate and overall length (Figure 4). These defects are corroborated *in-vivo* by both the high frequency of empty seed positions in the bottom of *ala6-1/7-2* siliques (Figure 2) and the 0% success rate of *ala6-1/7-2* pollen in competition with wild-type to fertilize ovules in the bottom third of a pistil (Table 3). Only a weak reproductive defect was observed for *ala6-1* pollen, indicating that ALA6 and ALA7 provide a significant level of redundancy (Table 1). The observation of a pollen-specific defect for *ala6-1/7-2* is consistent with mRNA expression profiling data, which indicate that both isoforms are expressed primarily in pollen (Figure S1b).

### The subcellular localization of ALA6 includes the plasma membrane and endomembrane structures

GFP-ALA6 and ALA6-YFP fusion proteins were observed both at the perimeter of pollen tubes, consistent with plasma membrane localization, and on highly mobile endomembrane structures (Figure 5, Movies S1, S2). While we cannot completely rule out the possibility of artifacts arising from over-expression or the fluorescent tags, identical localization patterns were observed at both high and low levels of protein expression, and for both N- and C-terminal tags. In addition, because GFP-ALA6 and ALA6-YFP fusion proteins rescued the *ala6-1/7-2* pollen defect, we conclude that at least a subset of the observed fusion proteins were in functional locations. This supports a working hypothesis that ALA6 and ALA7 might function in multiple membrane locations, including the plasma membrane.

### Lipid composition is altered in *ala6-1/7-2* pollen

Tandem mass spectroscopy revealed a ~2-fold increase in PA concentration and a ~2-fold decrease in PI concentration in *ala6-1/7-2* pollen grains relative to wild-type (Figure 6, File S1). While the yeast P4-ATPase mutants *Δdrs2* and *Δdrs2Δdnf1Δdnf2* have been linked to altered concentrations of PE, PS, PC, and PI (Pomorski et al., 2003), the lipid changes in *ala6-1/7-2* are distinct in two important ways. First, no change in PA concentration was observed for either yeast mutant, whereas a ~2-fold increase was observed for *ala6-1/7-2* pollen. Second, increased PI concentration was observed for the yeast mutant *Δdrs2Δdnf1Δdnf2*, whereas a ~2-fold decrease was observed for *ala6-1/7-2* pollen. A parallel lipid profiling analysis was not done on growing pollen tubes because of the difficulties in obtaining sufficient sample material. Therefore, we cannot exclude the possibility that the lipid profile of *ala6/7* pollen changes during tube growth. It is also possible that the observed differences between Col-0 and *ala6/7* pollen are the result of limited changes in lipid composition at a specific subcellular location.

While the mechanisms causing altered lipid concentrations in *ala6/7* pollen are currently unknown, the correlation between an increase in PA concentration and a decrease in PI concentration could be explained by changes in either the biosynthesis or degradation of PI. For example, loss of ALA6/7 might alter the properties of one or more membrane systems, and thereby indirectly inhibit the activities of enzymes involved in PI biosynthesis, such as ER-associated PI synthase (PIS) proteins (Collin et al., 1999; Justin et al., 2002; Löfke et al., 2008). An alternate and non-exclusive possibility is that loss of ALA6/7 might result in increased degradation of PI into PA through the activities of phospholipase C (PLC) and diacylglycerol kinase (DGK) (Testerink and Munnik, 2011). PA has a well-established role in stress signaling (Testerink and Munnik, 2005, 2011), and it is possible that PA production is increased in mutant pollen as a result of a “physiological stress condition” associated with the loss of ALA6/7. It is also possible that loss of ALA6/7 might disrupt a feedback mechanism that adjusts the levels of PI metabolites, which have been shown to regulate the activity of the yeast P4-ATPase Drs2p (Azouaoui et al., 2014).

### Loss of ALA6 and ALA7 disrupts FM dye staining of the pollen tube plasma membrane

A ~10-fold decrease in FM dye staining was observed for live *ala6-1/7-2* pollen tubes relative to wild-type (Figure 7). FM dyes selectively label membranes and are commonly used as tools for studying endocytosis and other vesicular trafficking processes (Betz et al., 1996; Bolte et al., 2004; Hoopmann et al., 2012). When a cell is exposed to an FM dye, the plasma membrane is stained immediately, followed by a time-dependent internalization into endomembrane structures (Bolte et al., 2004). While it is not clear how the loss of ALA6/7 reduces the ability of an FM dye to stain the PM, possible explanations include changes in the PM's surface charge or fluidity. However, it is unclear whether the PM's charge and fluidity could be altered enough to block FM dye staining without compromising the ability the membrane to carry out basic biological functions. Alternate explanations include quenching or sequestration of FM dyes by an unknown molecule(s) in the extracellular matrix or plasma membrane of mutant pollen tubes.

### Lipid transport activity of ALA6

In an attempt to determine the lipid transport activity of ALA6, the enzyme was expressed in the yeast triple mutant *Δdrs2Δdnf1Δdnf2* (Hua et al., 2002). This yeast mutant is deficient in phospholipid transport across the plasma membrane and has been successfully used to quantify the lipid transport activities of ALA2 and ALA3 (Poulsen et al., 2008; López-Marqués et al., 2010). However, ALA6 failed to show any lipid translocation activity for NBD-labeled fluorescent analogs of PS, PE, PC, and PA (Figure S5a). While controls corroborated that ALA6 was expressed at the yeast plasma membrane (Figure S5b), it remains unclear if ALA6 has a very different substrate specificity compared to other characterized flippases, or if the heterologous system was lacking a component that is uniquely required for ALA6 activity.

### Specialization of P4-ATPases

The P4-ATPase protein family in plants can be divided into five subfamilies (Baxter et al., 2003), each containing at least one pollen-expressed ALA isoform (Figure S1a). Of the seven pollen expressed ALA isoforms, three have been linked to pollen fertility: ALA3, subfamily 4 (Zhang and Oppenheimer, 2009; McDowell et al., 2013) and ALA6/7, subfamily 3 (this report). While decreased pollen fertility was observed for both *ala3* and *ala6/7* mutants, the underlying defects were distinct. For example, disorganized cytoplasmic streaming was observed in *ala3* pollen tubes (McDowell et al., 2013) but not *ala6/7* (Figure S6). Also, a change in lipid composition was observed in *ala6/7* pollen grains (Figure 6, File S1), but not *ala3* (McDowell et al., 2013). The differences between the *ala3* and *ala6/7* knockout phenotypes suggest that ALA3 and ALA6/7 have different cellular functions. Furthermore, the inability of the other four pollen-expressed ALA isoforms to compensate for the loss of ALA3 or ALA6/7 suggests that each ALA subfamily might have unique biological functions. The biological functions of the four other pollen-expressed ALA isoforms are currently unknown.

### Role of P4-ATPases in temperature stress tolerance

One of the most dramatic defects associated with *ala6-1/7-2* was hypersensitivity to temperature stress. While seed set was reduced to ~55% of wild-type under unstressed conditions (Figure 2), *ala6-1/7-2* plants were completely sterile under hot-day/cold-night temperature stress (Figure 3). In comparison, the seed set reduction in wild type plants was ~2-fold. Interestingly, temperature hypersensitivity has also been reported for *ala3* knockouts (McDowell et al., 2013) and a knockdown of *ALA1* (Gomès et al., 2000). In addition, the yeast P4-ATPase Drs2p is required for cell growth at or below 23°C (Ripmaster et al., 1993; Siegmund et al., 1998). It is not clear if these hypersensitivities are caused by defects in stress signaling, or biophysical differences resulting from changes in lipid composition or secretory pathway functions.

### Models for ALA6 and ALA7 in pollen tube growth

From the evidence presented in this study, we propose that ALA6 and ALA7 function to directly or indirectly change the distributions and concentrations of PA and PI. PA and the phosphorylated derivatives of PI (phosphatidylinositol phosphates, PIPs), have well-established roles as signaling molecules in plants, animals, and yeast (Wang, 2004; Stace and Ktistakis, 2006; Michell, 2008; Raghu et al., 2009; Ischebeck et al., 2010; Potocký et al., 2014). In plants, PA and PIPs are known to function as membrane-localization signals by binding specific protein targets, and can also regulate protein activity by inducing conformational changes (Wang et al., 2006; Munnik and Testerink, 2009; Xue et al., 2009; Munnik and Nielsen, 2011; Testerink and Munnik, 2011). Both PA and PIPs also have well-established roles in vesicular trafficking and pollen development (Thole and Nielsen, 2008; Fu, 2010; Ischebeck et al., 2010; Testerink and Munnik, 2011). For example, PA has been shown to affect the actin cytoskeleton of Arabidopsis pollen tubes via direct interaction with the actin capping protein AtCP (Huang et al., 2006). Altered PA levels result in disruptions to the pollen tube actin cytoskeleton and reduced tip growth (Potocký et al., 2003; Monteiro et al., 2005; Pleskot et al., 2013). Similarly, transient knockdown studies of NtPLDβ1, a PA-producing enzyme in tobacco, revealed a reduction in pollen tube growth that could be rescued by the addition of exogenous PA (Pleskot et al., 2010).

While it is not yet clear how the loss of ALA6 and ALA7 results in pollen tube growth defects, two non-exclusive models warrant consideration. First, the loss of ALA6 and ALA7 might result in the defective regulation of plasma membrane lipid asymmetry, which could compromise membrane-associated functions such as protein recruitment, localized membrane curvature, signaling, or membrane fluidity. For example, a reduced ability to flip PA or PI to the inner leaflet of the pollen tube plasma membrane might alter the ability of the PM to recruit proteins involved in exocytosis and polar cell growth. In a second model, loss of ALA6 and ALA7 might impair membrane curvature during the formation of endomembrane vesicles, thereby causing a general disruption of the secretory and endocytosis pathways. These pathways are of general importance for all cells, especially pollen tubes, which display one of the most rapid polar growth rates of any plant cell.

Regardless of mechanism, we show here that the P4-ATPases ALA6 and ALA7 are critical for rapid, sustained pollen tube growth as well as tolerance to a temperature stress. Together, these results support a model in which ALA6 and ALA7 have distinct activities from the five other pollen-expressed ALA proteins and directly or indirectly change membrane features important for pollen fitness.

### Conflict of interest statement

The authors declare that the research was conducted in the absence of any commercial or financial relationships that could be construed as a potential conflict of interest.

## Supplementary material

Supplementary Movie S 1 **Movie of NaAz-treated pollen tube expressing GFP-ALA6.** Movie depicts the pollen tube shown in Figure 5c. See caption to Figure 5 for details. Images were taken at regular intervals of 1.25 s over a 2 m time period. Movie plays at 15x speed.

Supplementary Movie S 2 **Movie of growing pollen tube expressing GFP-ALA6.** Movie depicts the pollen tube shown in Figure 5d. See caption to Figure 5 for details. Images were taken at regular intervals of 1.25 s over a 2 m time period. Movie plays at 15x speed.
