# Supplementary material for: Loss of the Arabidopsis thaliana P4-ATPases ALA6 and ALA7 impairs pollen fitness and alters the pollen tube plasma membrane
Source: Front Plant Sci. 2015 Apr 21;6:197. doi: 10.3389/fpls.2015.00197 (PMC4404812; doi:10.3389/fpls.2015.00197)
Supplement: Supplementary Movie S 1 — Movie of NaAz-treated pollen tube expressing GFP-ALA6. Movie depicts the pollen tube shown in Figure 5c. See caption to Figure 5 for details. Images were taken at regular intervals of 1.25 s over a 2 m time period. Movie plays at 15x speed. [file Presentation1.ZIP › Supplementary material/Figure S1.PDF]

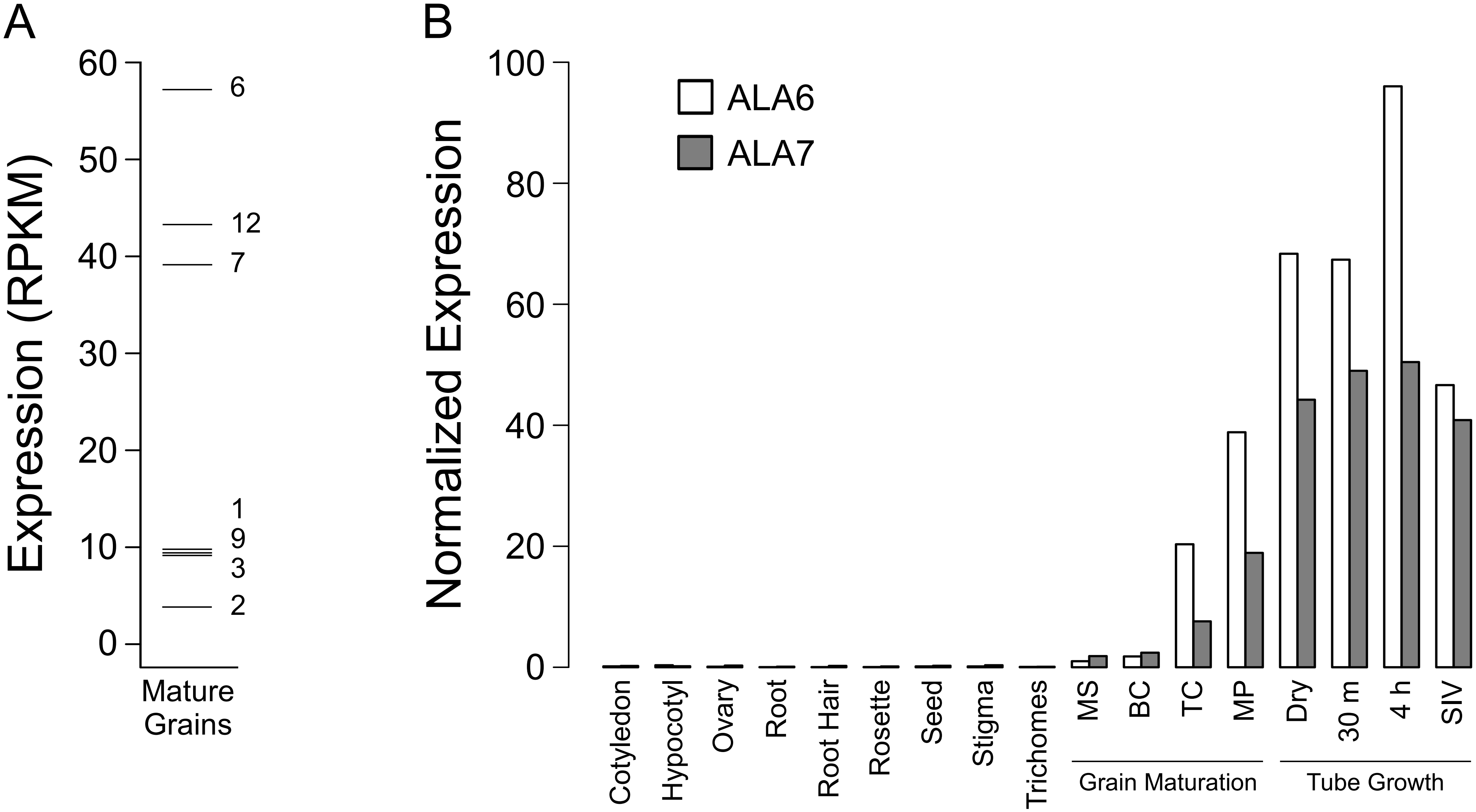

Figure S1. Expression profiling data showing preferential pollen expression for *ALA6* and *ALA7* mRNAs. (A) Expression levels of the seven pollen-expressed *ALA* isoforms in pollen grains: *ALA1* (At5g04930), *ALA2* (At5g44240), *ALA3* (At1g59820), *ALA6* (At1g54280), *ALA7* (At3g13900), *ALA9* (At1g68710), and *ALA12* (At1g26130). Expression data was obtained from a pollen RNA-seq dataset (Loraine et al., 2013) and is expressed as reads per KB per million reads (RPKM). (B) Expression of *ALA6* and *ALA7* in different tissues. Data was obtained from the Arabidopsis eFP Browser (http://bar.utoronto.ca/efp/cgi-bin/efpWeb.cgi) (Winter et al., 2007) and was normalized against: *EF1-alpha* (AT5G60390), *CBP20* (At5g44200), *Actin-2* (At3g18780), and *UBC* (At5g25760). Expression of *ALA6* in microspore (MS) tissue was arbitrarily set to 1, and the rest of the data adjusted accordingly. Expression data for pollen grain maturation (Honys and Twell, 2004) and pollen tube growth (Qin et al., 2009) were collected in independent experiments. Developmental stages are abbreviated as: MS, microspore; BC, bicellular pollen; TC, tricellular pollen; MP, mature pollen; 30 m, pollen tube germinated *in-vitro* for 30 minutes; 4 h, pollen tube germinated *in-vitro* for 4 hours; and SIV, pollen tubes after semi *in-vivo* growth through a stigma.
