# Supplementary material for: Loss of the Arabidopsis thaliana P4-ATPases ALA6 and ALA7 impairs pollen fitness and alters the pollen tube plasma membrane
Source: Front Plant Sci. 2015 Apr 21;6:197. doi: 10.3389/fpls.2015.00197 (PMC4404812; doi:10.3389/fpls.2015.00197)
Supplement: Supplementary Movie S 1 — Movie of NaAz-treated pollen tube expressing GFP-ALA6. Movie depicts the pollen tube shown in Figure 5c. See caption to Figure 5 for details. Images were taken at regular intervals of 1.25 s over a 2 m time period. Movie plays at 15x speed. [file Presentation1.ZIP › Supplementary material/Figure S2.PDF]

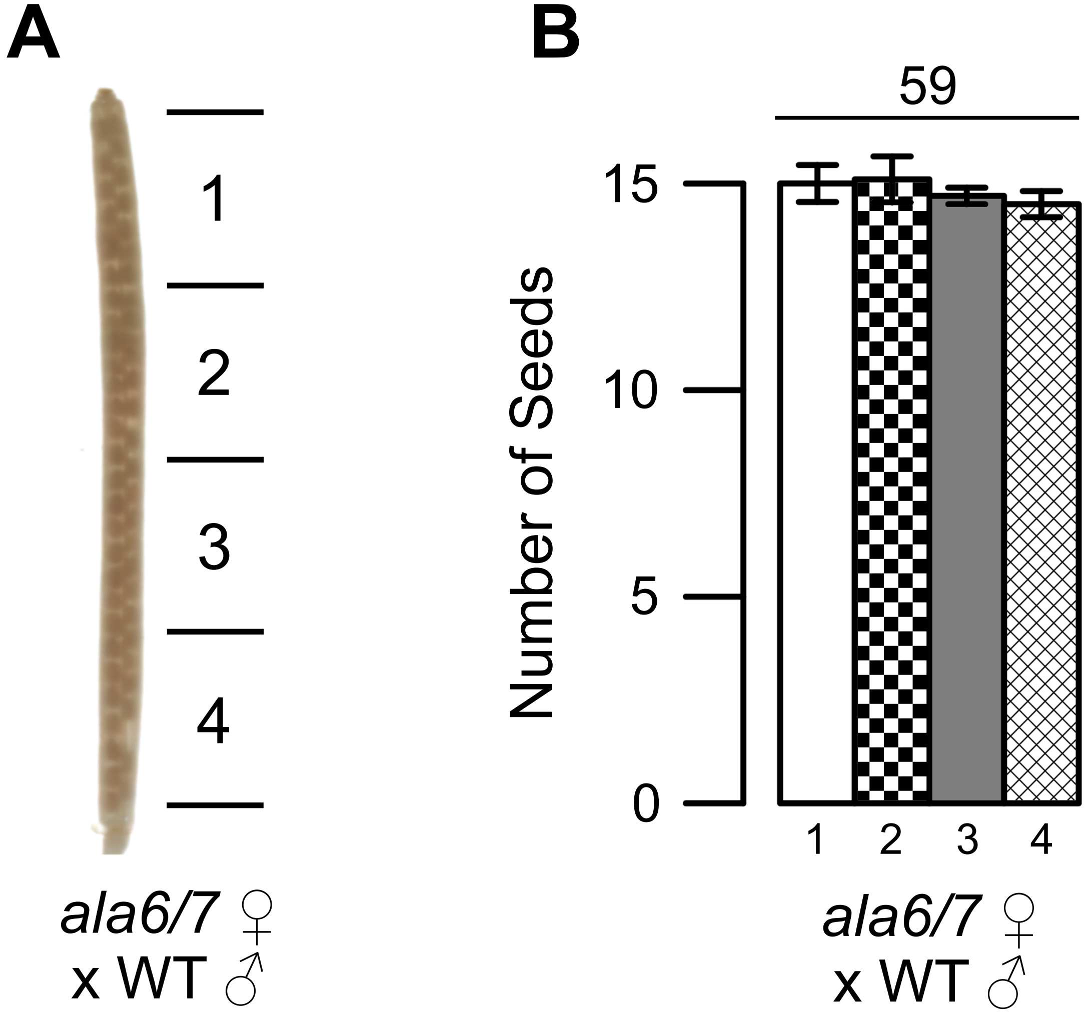

Figure S2. Fertilization of *ala6-1/7-2* pistils with wild-type pollen resulted in siliques with full seed set. (A) Representative example of an *ala6-1/7-2* silique fertilized with wild-type (WT) pollen and cleared with 70% EtOH to show seed positions. Siliques were divided into four sectors of equal length with sector 1 at the top (stigma end) of the silique and sector 4 at the base of the silique. Sector numbers appear to the right of the silique. (B) Graph of seed set by sector. Average results (±SE) are reported for two independent experiments; n = 6 siliques, each from a different plant. Sector numbers appear below each column and the average total seed set is given above the sector data.
