# Supplementary material for: Loss of the Arabidopsis thaliana P4-ATPases ALA6 and ALA7 impairs pollen fitness and alters the pollen tube plasma membrane
Source: Front Plant Sci. 2015 Apr 21;6:197. doi: 10.3389/fpls.2015.00197 (PMC4404812; doi:10.3389/fpls.2015.00197)
Supplement: Supplementary Movie S 1 — Movie of NaAz-treated pollen tube expressing GFP-ALA6. Movie depicts the pollen tube shown in Figure 5c. See caption to Figure 5 for details. Images were taken at regular intervals of 1.25 s over a 2 m time period. Movie plays at 15x speed. [file Presentation1.ZIP › Supplementary material/Figure S3.PDF]

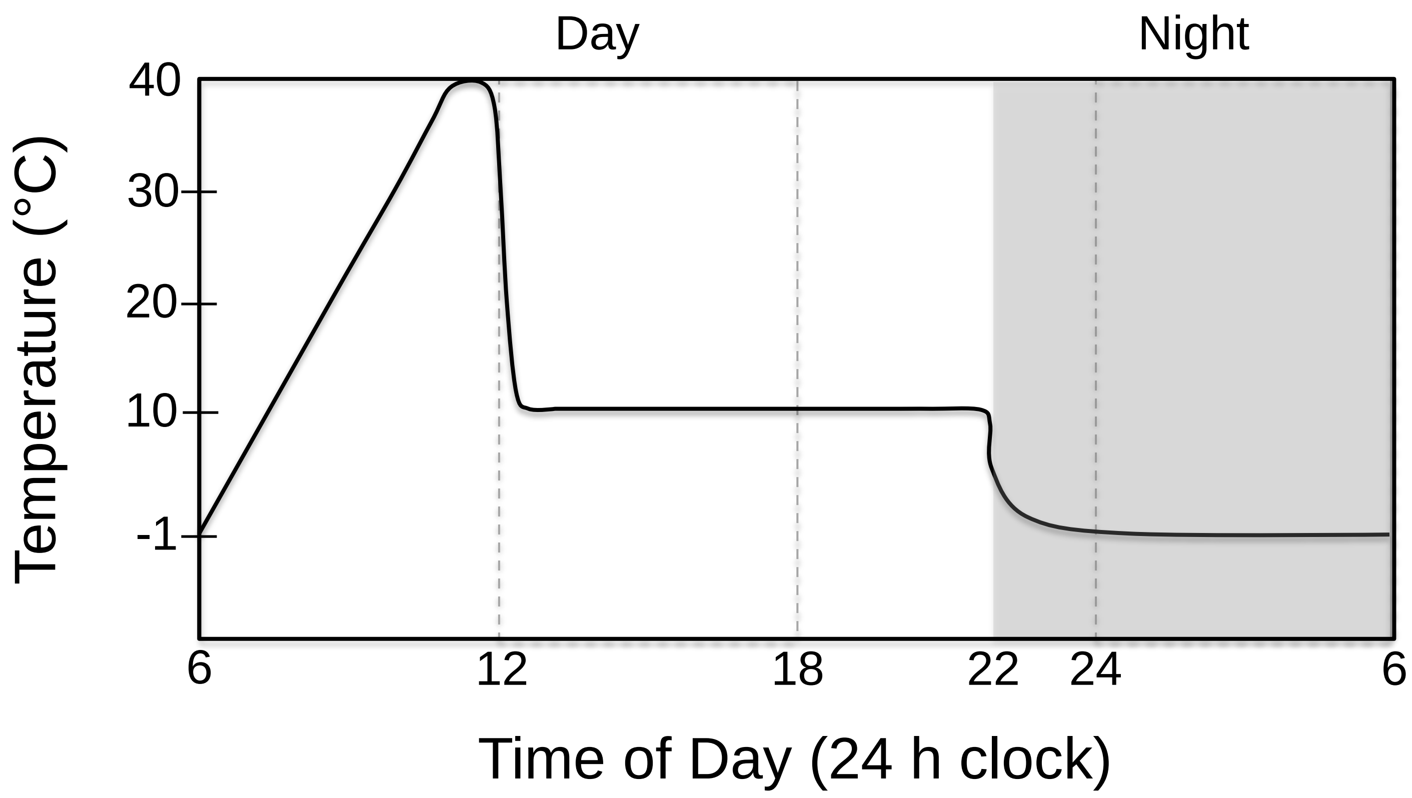

Figure S3. Schematic diagram of the hot-day/cold-night temperature stress that blocked pollen fertility in *ala6-1/7-2* plants. Temperature cycles from 40°C during the day to -1°C at night, with periods of intermediate temperature between the extremes for acclimation. Manually pollinated plants were immediately moved to hot-day/cold-night stress between 15:00 and 17:00 h on the diurnal cycle (chamber temperature of 10°C), forcing the period of pollen tube growth and fertilization to overlap with stress temperatures.
