# Supplementary material for: Loss of the Arabidopsis thaliana P4-ATPases ALA6 and ALA7 impairs pollen fitness and alters the pollen tube plasma membrane
Source: Front Plant Sci. 2015 Apr 21;6:197. doi: 10.3389/fpls.2015.00197 (PMC4404812; doi:10.3389/fpls.2015.00197)
Supplement: Supplementary Movie S 1 — Movie of NaAz-treated pollen tube expressing GFP-ALA6. Movie depicts the pollen tube shown in Figure 5c. See caption to Figure 5 for details. Images were taken at regular intervals of 1.25 s over a 2 m time period. Movie plays at 15x speed. [file Presentation1.ZIP › Supplementary material/Figure S4.PDF]

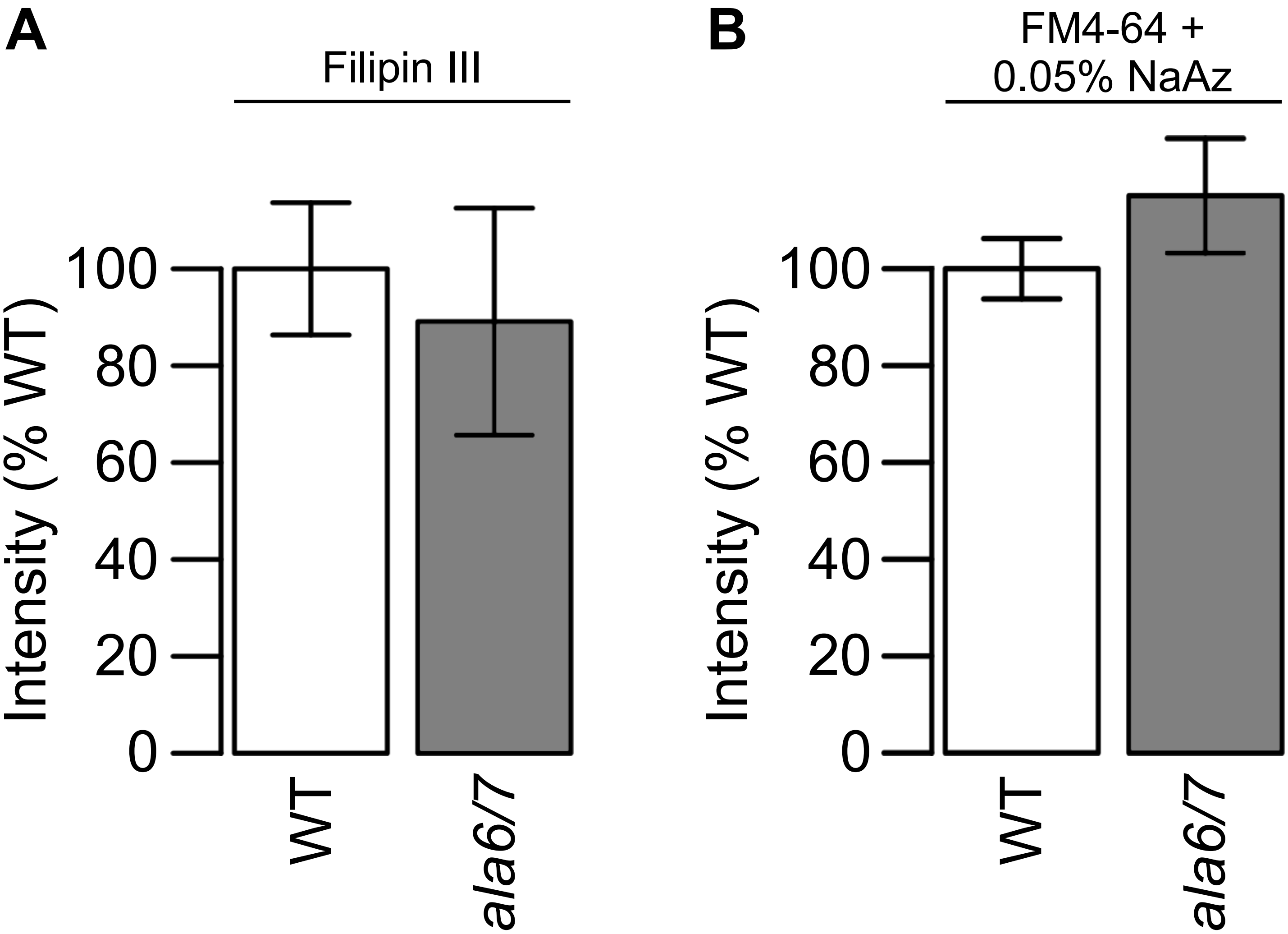

Figure S4. Loss of ALA6 and ALA7 does not reduce the staining of membranes by Filipin, or the ability of FM dyes to stain pollen tubes after NaAz Treatment. (A) Filipin III dye staining of living pollen tubes. Average results (±SE) are reported for two independent experiments, n = 8 pollen tubes for both genotypes. (B) FM4-64 staining of pollen tubes killed by prolonged exposure to 0.05% NaAz. Average results (±SE) are reported for three independent experiments, n = 9 pollen tubes for *ala6-1/7-2* and n = 25 pollen tubes for wild-type. Pollen tubes were collected from four or more different plants for each genotype and dye combination. Differences between wild-type and *ala6-1/7-2* were not statistically significant (p=0.705 for Filipin III and p=0.337 for FM4-64, Welch's t-test).
