# Supplementary material for: Loss of the Arabidopsis thaliana P4-ATPases ALA6 and ALA7 impairs pollen fitness and alters the pollen tube plasma membrane
Source: Front Plant Sci. 2015 Apr 21;6:197. doi: 10.3389/fpls.2015.00197 (PMC4404812; doi:10.3389/fpls.2015.00197)
Supplement: Supplementary Movie S 1 — Movie of NaAz-treated pollen tube expressing GFP-ALA6. Movie depicts the pollen tube shown in Figure 5c. See caption to Figure 5 for details. Images were taken at regular intervals of 1.25 s over a 2 m time period. Movie plays at 15x speed. [file Presentation1.ZIP › Supplementary material/Figure S5.PDF]

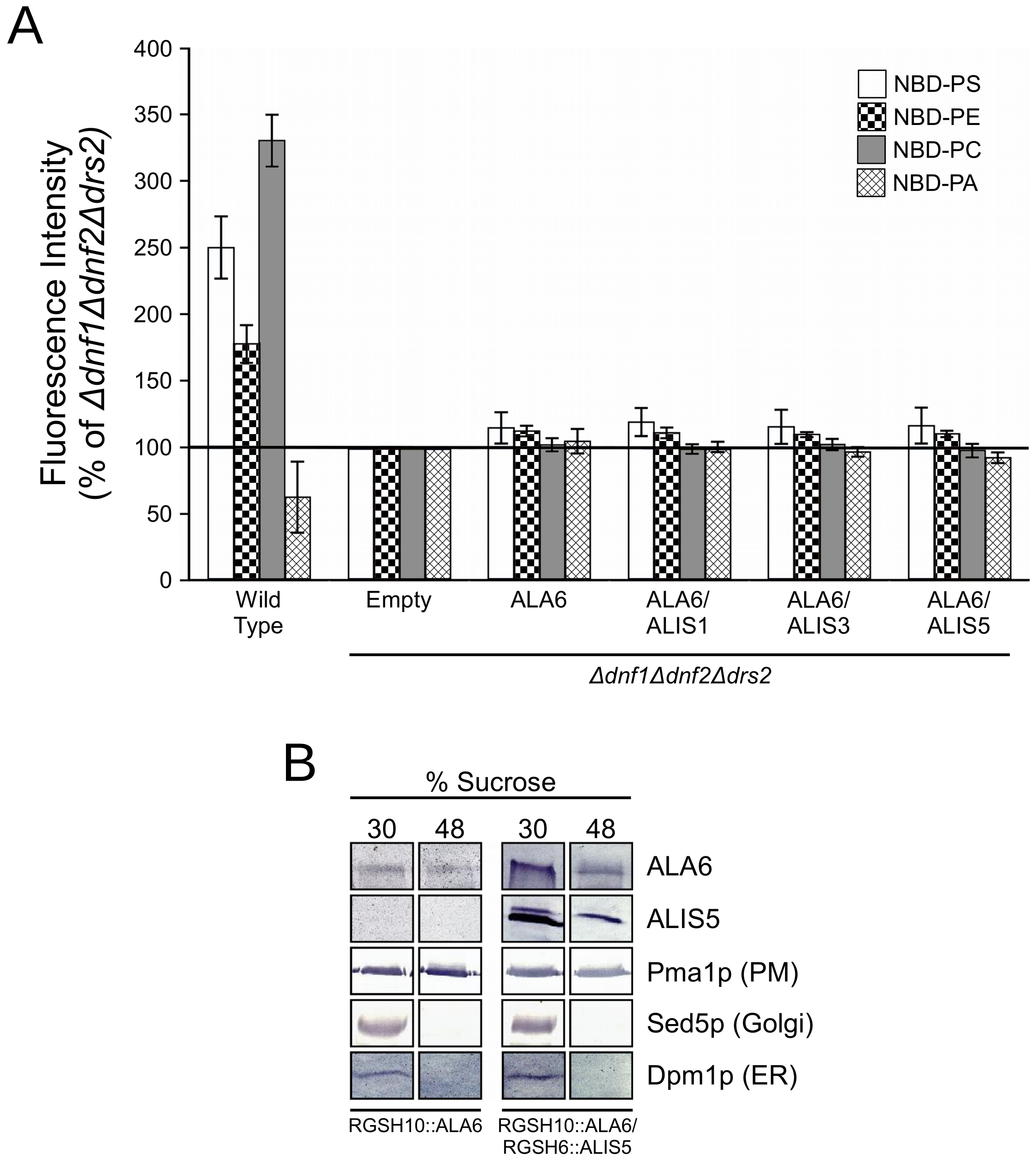

Figure S5. Expression of ALA6 in yeast failed to show detectable flippase activity. (A) Lipid translocation assays were carried out for ALA6 alone and in the presence of three putative beta-subuits: ALIS1, ALIS3, and ALIS5. An empty vector was used as a negative control, and vectors were transformed into wild type yeast as a positive control. Average results (±SE) are reported for at least four independent experiments. (B) Discontinuous sucrose density gradient fractionation of PM-enriched yeast membranes expressing RGSH10:ALA6 alone or coexpressed with RGSH6:ALIS1. Fractions enriched in ER (30% sucrose) and PM (48% sucrose) were analyzed with a Western blot using the following antibodies: anti-Pma1p, plasma membrane; anti-Dpm1p, ER; anti-Sed5p, Golgi; anti-RGSHis, ALA6 and ALIS5.
