# Supplementary material for: Loss of the Arabidopsis thaliana P4-ATPases ALA6 and ALA7 impairs pollen fitness and alters the pollen tube plasma membrane
Source: Front Plant Sci. 2015 Apr 21;6:197. doi: 10.3389/fpls.2015.00197 (PMC4404812; doi:10.3389/fpls.2015.00197)
Supplement: Supplementary Movie S 1 — Movie of NaAz-treated pollen tube expressing GFP-ALA6. Movie depicts the pollen tube shown in Figure 5c. See caption to Figure 5 for details. Images were taken at regular intervals of 1.25 s over a 2 m time period. Movie plays at 15x speed. [file Presentation1.ZIP › Supplementary material/Figure S6.PDF]

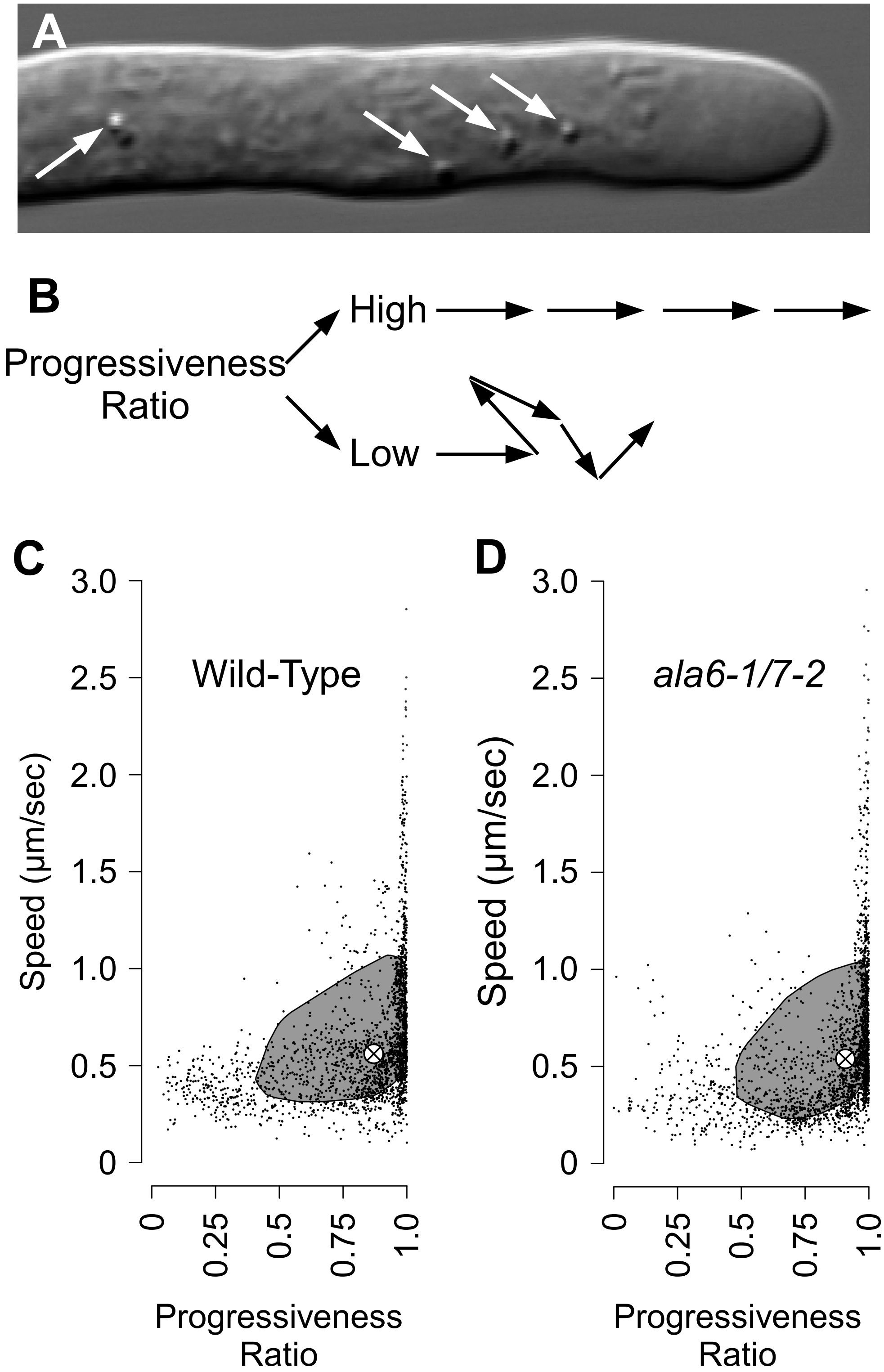

Figure S6. Cytoplasmic streaming is not altered in *ala6-1/7-2* pollen tubes. (A) Wild-type pollen tube with white arrows pointing to representative vesicles visible with DIC optics. Scale bar = 5µM. (B) Exemplary model trajectories with high and low progressiveness ratios. (C and D) Bagplots (Rousseeuw et al., 1999) of vesicular speed and progressiveness ratios for vesicles within wild-type (C) and *ala6-1/7-2* (D) pollen tubes. The shaded region represents an area containing 50% of the data points. The two-dimensional median is represented by the crosshairs within the shaded region. Differences between wild-type and *ala6-1/7-2* were not statistically significant for either speed (p=0.923) or progressiveness ratio (p=0.444), Welch's t-test. N = 4 for both wild-type and *ala6-1/7-2*.
